# Transcriptomic Analyses of Root Restriction Effects on Phytohormone Content and Signal Transduction during Grape Berry Development and Ripening

**DOI:** 10.3390/ijms19082300

**Published:** 2018-08-06

**Authors:** Feng Leng, Jinping Cao, Shiping Wang, Ling Jiang, Xian Li, Chongde Sun

**Affiliations:** 1 Laboratory of Fruit Quality Biology/The State Agriculture Ministry Laboratory of Horticultural Plant Growth, Development and Quality Improvement, Zijingang Campus, Zhejiang University, Hangzhou 310058, China; lengfeng.214@163.com (F.L.); caojinpingabc@126.com (J.C.); xianli@zju.edu.cn (X.L.); 2 College of Horticulture and Plant Protection, Yangzhou University, Yangzhou 225009, China; 3 School of Agriculture and Biology, Shanghai Jiao Tong University, Shanghai 200240, China; fruit@sjtu.edu.cn; 4 Wujiang Research Institute of Grape, Jinhua 321017, China; jlonly@sina.com

**Keywords:** root restriction, grape development, phytohormones, RNA-Seq

## Abstract

Phytohormones strongly influence growth, development and nutritional quality of agricultural products by modulating molecular and biochemical changes. The purpose of the present study was to investigate the influence of root restriction (RR) treatment on the dynamic changes of main phytohormones during the berry development and ripening of “Summer Black” early ripening seedless grape (*Vitis vinifera* × *V. labrusca*), and to analyze the changes in the biosynthesis and signal transduction pathways of phytohormones by transcriptomics. Enzyme-linked immunosorbent assay (ELISA) and Ultra Performance Liquid Chromatography-High Resolution Mass Spectrometry (UPLC-HRMS) were used to quantify the phytohormone levels, and RNA-Seq was used to analyze the transcript abundance. The results showed that 23 transcripts involved in the phytohormone biosynthesis and 34 transcripts involved in the signal transduction pathways were significantly changed by RR treatment. RR also increased abscisic acid, brassinosteroid, ethylene, jasmonic acid and salicylic acid levels, while decreasing auxin, cytokinin, and gibberellin contents. The results of the present study suggest that RR treatment can accelerate the grape ripening process, and specific candidate genes were identified for further functional analysis.

## 1. Introduction

Grape is a non-climacteric fruit of great economic importance for both fresh produce and wine production. The berries undergo a complex series of physiological and molecular changes during development and ripening [1]. Plants have a sessile lifestyle and respond to numerous external stimuli and coordinate their growth and development accordingly [2]. These adaptations include responses to temperature and water stresses, nutrients and pathogens, many of which are mediated by phytohormones, a group of structurally unrelated small molecules, which include auxin, cytokinine, gibberellin, abscisic acid, ethylene, brassinosteroid, jasmonic acid and salicylic acid [2].

Root restriction (RR) is a cultivation technique where roots are controlled in a certain volume to restrict their growth and to improve the use of agricultural resources [3,4]. Previous reports demonstrated that RR treatment limits the growth of shoots, increases the sugars and anthocyanin contents and enhances the nitrate uptake rate of berries [3,4,5,6,7]. Phytohormones play important roles in the fruit development, and the biotic and abiotic stresses responses [1,2]. However, the changes in endogenous phytohormones in grape berries in response to the RR treatment are still poorly understood. A better understanding of dynamic changes of phytohormones at the molecular level will help to improve the way RR is performed. Thus, we analyzed the changes of major phytohormones and the genes involved in their biosynthesis and signal transduction pathways during fruit development and ripening using “Summer Black” grape berry, aiming to explore the effects of RR treatment on phytohormones in the berry fruits at the molecular level.

## 2. Results

### 2.1. Changes in Phytohormone Content during Grape Berry Development

**Figure 1:**
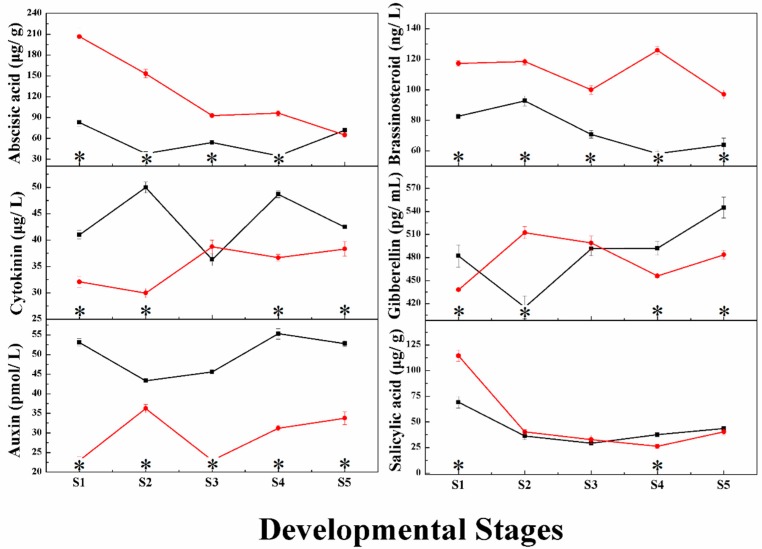
The changes of phytohormone contents during grape berry development and ripening under RR treatment (red line) and control (black line). * indicates the significant differences with *t*-test (*p* < 0.05, *n* = 3).

The differences in grape berry endogenous phytohormones between the control and RR treatments during fruit development are shown in Figure 1. The auxin contents in RR treatment were significantly lower than in the control treatment throughout development and ripening, while the brassinosteroid contents showed the opposite trend, with the contents of brassinosteroid in the RR treatment significantly higher than in the control group. The cytokinin contents in the RR treatment were significantly lower than those in the control group at S1, S2, S4 and S5 stages, and the gibberellin contents in the RR treatment were significantly higher at S2 stage but lower at S1, S4 and S5 stages than in the control group. No significant difference in cytokinin and gibberellin contents was seen between the two groups at the S3 stage. The detection and quantification of abscisic acid and salicylic acid were carried out by UPLC-HRMS. The level of abscisic acid and salicylic acid were relatively high at the fruitlet stage, and then declined during fruit development and ripening, with a slight increase at the fully ripe stage. Grapes from plants in the RR treatment had higher abscisic acid at S1, S2, S3 and S4 stages than the control group. For salicylic acid, its content in the RR treatment was significantly higher at S1 stage and lower at S4 stage compared to that of the controls.

### 2.2. RNA-Sequencing Results

RNA-Seq was adopted for comparative transcriptome analysis of grape berries in response to RR treatment during grape development and ripening, to generate expression profiles for both treatments, with three independent biological replicates for each. The sequence reads were matched to the Pinot Noir 40024 reference genome [8]. The mapped ratio was about 60–70%, After aligning and assembling, the expression of 29,971 genes was detected by removal of partial overlapping sequences [9]. A quantitative evaluation of the transcripts was used to measure the levels of differential expression of genes related to the phytohormone biosynthesis and signal transduction pathways between the RR and control groups through all developmental stages. Differences in expression were considered as significant with the false discovery rate < 0.05 and Log_2_|fold change| > 1. A total of 57 transcripts (23 involved in hormone biosynthesis pathways and 34 involved in the signaling pathways) were significantly influenced by RR treatment at different growth stages (Table 1). The impacts of RR treatment on the berry were the greatest at the young stage, following by the veraison stage. Interestingly, no transcript was influenced by RR treatment at the fully ripe stage.

### 2.3. Phytohormone Biosynthesis and Signal Transduction Pathways

#### 2.3.1. Auxin

**Figure 2:**
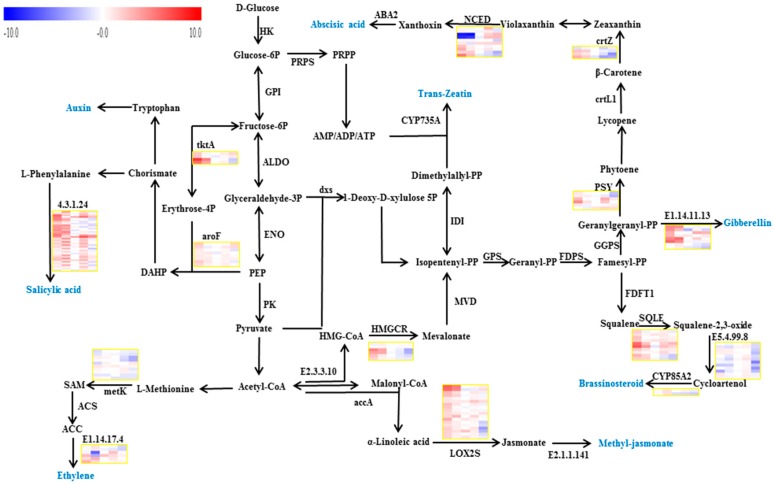
The phytohormone biosynthesis pathways in grape berry. Boxes from left to right follow the berry development. The data set was normalized to the values at the before veraison stage in the control treatment as log2 transformed, and the relative expression changes at the other treatment and other stages in the relation to the before veraison stage in the control treatment were hence expressed as log2 fold change, the upper set of boxes is for the control treatment and the lower set is for the RR treatment. PEP, Phosphoenolpyruvate; DAHP, 2-Dehydro-3-deoxy-d-arabino-heptonate 7-phosphate; SAM, *S*-Adenosyl-l-methionine; ACC, 1-Aminocyclopropane-1-carboxylate; PRPP, 5-Phosphoribosyl 1-pyrophosphate; HMGCR, hydroxymethylglutaryl-CoA reductase; LOX2S, lipoxygenase; NCED, 9-*cis*-epoxycarotenoid dioxygenase; CYP85A2, brassinosteroid-6-oxidase 2; E1.14.11.13, gibberellin 2-oxidase; crtZ, beta-carotene 3-hydroxylase; SQLE, squalene monooxygenase; E1.14.17.4, aminocyclopropanecarboxylate oxidase; E2.1.1.141, jasmonate O-methyltransferase; tktA, transketolase; dxs, 1-deoxy-d-xylulose-5-phosphate synthase; E2.3.3.10, hydroxymethylglutaryl-CoA synthase; GPS, geranyl diphosphate synthase; metK, S-adenosylmethionine synthetase; FDPS, farnesyl-diphosphate synthase; FDFT1, farnesyl-diphosphate farnesyltransferase; GGPS, geranylgeranyl diphosphate synthase; PSY, phytoene synthase; aroF, 3-deoxy-7-phosphoheptulonate synthase; PRPS, ribose-phosphate pyrophosphokinase; MVD, diphosphomevalonate decarboxylase; E4.3.1.24, phenylalanine ammonia-lyase; ACS, 1-aminocyclopropane-1-carboxylate synthase; IDI, isopentenyl-diphosphate delta-isomerase; E5.4.99.8, cycloartenol synthase; crtL1, lycopene beta-cyclase; accA, acetyl-CoA carboxylase carboxyl transferase subunit alpha; CYP735A, cytokinin trans-hydroxylase; HK, hexokinase; PK, pyruvate kinase; ALDO, fructose-bisphosphate aldolase; ENO, enolase; aceF, dihydrolipoamide acetyltransferase; GPI, glucose-6-phosphate isomerase.

**Figure 3:**
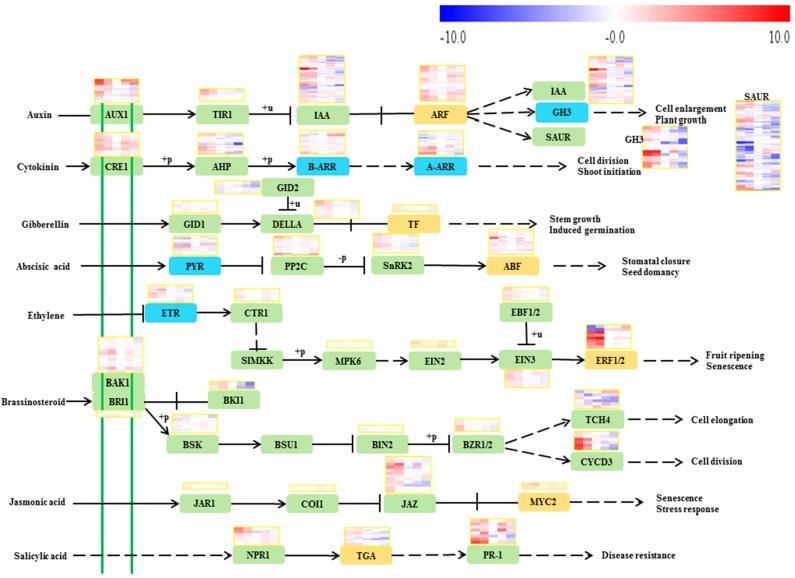
The phytohormone signaling transduction pathways in grape berry. Boxes from left to right follow the berry development. The data set was normalized to the values at the before veraison stage in the control treatment as log2 transformed, and the relative expression changes at the other treatment and other stages in the relation to the before veraison stage in the control treatment were hence expressed as log2 fold change, the upper set of boxes is for the control treatment and the lower set is for the RR treatment. A-ARR, two-component response regulator ARR-A family; B-ARR, two-component response regulator ARR-B family; ABF, ABA responsive element binding factor; AHP, histidine-containing phosphotransfer peotein; ARF, auxin response factor; AUX1, auxin influx carrier; BAK1, brassinosteroid insensitive 1-associated receptor kinase 1; BIN2, protein brassinosteroid insensitive 2; BKI1, BRI1 kinase inhibitor 1; BRI1, protein brassinosteroid insensitive 1; BSK, BR-signaling kinase; BZR1_2, brassinosteroid resistant 1/2; COI1, coronatine-insensitive protein 1; CRE1, cytokinin receptor; CTR1, serine/threonine-protein kinase CTR1; CYCD3, cyclin D3; DELLA, DELLA protein; EBF1_2, EIN3-binding F-box protein; EIN2, ethylene-insensitive protein 2; EIN3, ethylene-insensitive protein 3; ERF1, ethylene-responsive transcription factor 1; ETR, ethylene receptor; GH3, auxin-responsive GH3 gene family; GID1, gibberellin receptor GID1; GID2, F-box protein GID2; IAA, auxin-responsive protein IAA; JAR1, jasmonic acid-amino synthetase; JAZ, jasmonate ZIM domain-containing protein; MPK6, mitogen-activated protein kinase 6; MYC2, transcription factor MYC2; NPR1, regulatory protein NPR1; PP2C, protein phosphatase 2C; PR1, pathogenesis-related protein 1; PYR, abscisic acid receptor PYR family; SAUR, SAUR family protein; SNRK2, serine/threonine-protein kinase SRK2; TCH4, xyloglucan:xyloglucosyl transferase TCH4; TF, phytochrome-interacting factor 4; TGA, transcription factor TGA.

Auxin is synthesized from precursors generated via the shikimate pathway [10]. Endogenous auxin is transported to specific tissues to trigger the signaling cascades that causing developmental responses. Auxin-responsive genes fit into three major classes: auxin-responsive protein IAA, auxin-responsive GH3 gene family and SAUR gene families [11]. We found a transcript *VIT_15s0048g00370* coding for transketolase involved in the biosynthesis of chorismate was significantly down-regulated at the S1 and S2 stages by RR treatment, and this was in accordance with altered auxin levels in berries at the earlier stages of the two treatments, may play an important role in the control of auxin concentrations response to RR treatment. Conversely the transcript *VIT_00s0391g00070*, a gene involved in the biosynthesis of chorismate was up-regulated by RR treatment at the S4 stage (Figure 2). In the auxin signaling, RR treatment down-regulated the expression of the *VIT_07s0005g00090*, a transcript in auxin-responsive GH3 gene family, at the S1 stage. Eight transcripts coding for auxin-responsive protein IAA were significantly changed by RR treatment, with *VIT_05s0020g04670*, *VIT_05s0020g04680*, *VIT_05s0049g01970* and *VIT_09s0002g04080* were down-regulated at the S4 stage, *VIT_11s0016g04490* was up-regulated at the S1 stage and down-regulated at the S4 stage, *VIT_07s0141g00270* and *VIT_09s0002g05160* were up-regulated at the S1 stage respectively. Four transcripts coding for SAUR family protein were significantly changed by RR treatment, with three of them (*VIT_04s0023g00580*, *VIT_15s0048g00530* and *VIT_16s0098g01150*) up-regulated at the S1 stage, and the other one up-regulated at the S1 stage but down-regulated at the S2 stage during development and ripening (Figure 3).

#### 2.3.2. Cytokinin

Trans-zeatin is found to be the predominant cytokinin of grape berry, which is derived from the mevalonate pathway [12]. Cytokinin signaling is mediated by a multistep phosphorelay, which is comprised of cytokinin receptor, histidine-containing phosphotransfer peotein, type-B and type-A response regulators [13]. We found only one transcript *VIT_04s0044g01740* coding for hydroxymethylglutaryl-CoA reductase was down-regulated by RR treatment at the S1 stage in the trans-zeatin biosynthesis, which was in accordance with the changes of trans-zeatin content (Figure 2). In the cytokinin signaling, A-ARRs act as repressors of cytokinin-activated transcription. We found three transcripts *VIT_01s0026g00940*, *VIT_08s0007g05390* and *VIT_13s0067g03070* were up-regulated by RR treatment at the S1 stage, which were opposite to the decrease in trans-zeatin (Figure 3).

#### 2.3.3. Gibberellin

The synthesis of gibberellins mainly involves several kinds of enzymes, including geranyl diphosphate synthase, farnesyl-diphosphate synthase, geranylgeranyl diphosphate synthase and gibberellin 2-oxidase [14]. The gibberellin signal transduction pathway is consisting of gibberellin-insensitive dwarf 1, DELLA protein, gibberellin-insensitive dwarf 2 and phytochrome-interacting factor 4 [15,16]. We found the transcript *VIT_19s0140g00120* coding for gibberellin 2-oxidase, enzymes involved in the negative-feedback regulation in the bioactive GA-synthesis pathway, were up-regulated at the S1 stage and down-regulated at the S2 stage by RR treatment, which is negatively correlated with the gibberellin contents. On the other hand, a transcript encoding gibberellin receptor GID1 displayed significantly increased transcript abundance at the S1 stage by RR treatment (Figure 3).

#### 2.3.4. Abscisic Acid

Abscisic acid is synthesized from carotenoid precursor [17]. The signal transduction consists of pyrabactin (PYR) resistance, protein phosphatase 2c protein, sucrose nonfermenting-related kinase 2 and abscisic acid-responsive element binding factors [18,19]. Three transcripts coding for 9-cis-epoxycarotenoid dioxygenase were up-regulated (*VIT_19s0093g00550* at the S1 stage and *VIT_10s0003g03750* at the S4 stage), or down-regulated (*VIT_02s0087g00910* at the S4 stage) by RR treatment during growth processes (Figure 2). In addition, one transcript *VIT_02s0012g01270* coding for PYR was down-regulated at the S1 and S2 stages, one transcript *VIT_18s0001g10450* coding for ABA responsive element binding factor was up-regulated at the S1 stage, three transcripts *VIT_06s0004g05460*, *VIT_11s0016g03180* and *VIT_13s0019g02200* coding for protein phosphatase 2c protein were up-regulated at the S1 stage, and one transcript VIT_16s0050g02680 coding for protein phosphatase 2c protein was up-regulated at the S1, S3 and S4 stages by RR treatment in the abscisic acid signaling pathway (Figure 3).

#### 2.3.5. Ethylene

The biosynthesis of ethylene begins with the production of S-adenosylmethionine (SAM) which is catalyzed by SAM synthetase from L-methionine. SAM is then metabolized to 1-aminocyclopropane-1-carboxylic acid (ACC) by ACC synthase. Finally, ACC is oxidized by ACC oxidase to yield ethylene and CO_2_ [20]. Ethylene signal transduction involves ethylene receptor, serine/threonine-protein kinase CTR1, mitogen-activated protein kinase 4, mitogen-activated protein kinase 6, ethylene-insensitive protein 2, ethylene-insensitive protein 3 and ethylene-responsive transcription factor [21]. In our results, we observed one transcript *VIT_08s0007g05000* coding for SAM synthase was up-regulated at the S1 stage. Three transcripts coding for ACC oxidase was affected by RR treatment, with the transcript *VIT_11s0016g02380* up-regulated significantly at the S1 and S3 stages, and the transcript *VIT_12s0059g01380* just up-regulated significantly at the S1 stage, but other transcripts were not influenced by RR treatment (Figure 2). In addition, we found 5 transcripts involved in the ethylene signaling pathway were affected by RR treatment, in which the *VIT_06s0004g05240* and *VIT_05s0049g00090* coding for ethylene receptor were down-regulated at the S2 stage, the *VIT_05s0049g00510* was down-regulated at the S2 stage and up-regulated at the S3 stage, the *VIT_07s0005g03230* was down-regulated at the S1 and S2 stages, and the *VIT_07s0005g03260* was down-regulated at the S1 and S2 stages (Figure 3).

#### 2.3.6. Brassinosteroid

Brassinosteroids are biosynthesized from squalene: the squalene is converted to squalene-2,3-oxide by squalene monooxygenase, then via multiple steps into campesterol. Cytochrome P450 enzymes of CYP90 and CYP85 families catalyzing the last step from campesterol to brassinosteroid are negatively controlled by brassinosteroid at the transcriptional level [22,23]. Brassinosteroids signal transduction involves brassinosteroid insensitive 1, brassinosteroid insensitive 1-associated receptor kinase 1, brassinosteroid-signaling kinase, serine/threonine-protein phosphatase BSU1, brassinosteroid insensitive 1 kinase inhibitor 1, brassinosteroid insensitive 2, brassinosteroid resistant, xyloglucosyl transferase TCH4 and cyclin D3 [24]. We found the transcript *VIT_09s0054g01090* coding for cycloartenol synthase and the transcript *VIT_03s0088g01150* coding for squalene monooxygenase were down-regulated at the S1 stage, and the transcript *VIT_14s0083g01110* coding for brassinosteroid-6-oxidase 2 was down-regulated at the S4 stage by RR treatment in the brassinosteroid biosynthesis pathway (Figure 2). One transcript involved in brassinosteroid-signaling *VIT_11s0052g01190* coding for TCH4 was up-regulated at the S1 and S3 stages, but down-regulated at the S2 and S4 stages by RR treatment (Figure 3).

#### 2.3.7. Jasmonic Acid

Jasmonic acid and its volatile methyl ester (MeJA) are synthesized from linolenic acid by oxygenation with lipoxygenase [25]. Current models of jasmonic acid signal transduction involve jasmonic acid-amino synthetase, coronatine-insensitive protein 1, jasmonate ZIM domain-containing protein and transcription factor MYC2 [26,27]. We found one transcript *VIT_06s0004g01510* coding for lipoxygenase was up-regulated at the S4 stage, but another transcript *VIT_09s0002g01080* coding for lipoxygenase was up-regulated at the S1 stage and down-regulated at the S2 stage by the RR treatment in the jasmonic acid biosynthesis pathway (Figure 2). There are several different lipoxygenase genes, they have similar catalytic activities but may be involved in different branches pathway, leading to different products. On the other hand, three transcripts *VIT_01s0146g00480*, *VIT_09s0002g00890* and *VIT_11s0016g00710* coding for jasmonate ZIM domain-containing protein were up-regulated at the S1 stage by RR treatment in the jasmonic acid signaling pathway (Figure 3).

#### 2.3.8. Salicylic Acid

Salicylic acid is a product of phenylpropanoid metabolism formed via decarboxylation of trans-cinnamic acid to benzoic acid and its subsequent 2-hydroxylation to salicylic acid [28]. The salicylic acid signal transduction pathway contains regulatory protein NPR1, transcription factor TGA and pathogenesis-related protein 1 [29]. We found five transcripts *VIT_06s0004g02620*, *VIT_13s0019g04460*, *VIT_16s0039g01100*, *VIT_16s0039g01110* and *VIT_16s0039g01120* coding for phenylalanine ammonia-lyase were down-regulated at the S1 and S2 stages or up-regulated at the S3 and S4 stages by RR treatment respectively (Figure 2). In addition, one transcript *VIT_03s0088g00710* coding for pathogenesis-related protein 1 was up-regulated at the S1 and S4 stages, and another transcript *VIT_03s0088g00810* coding for pathogenesis-related protein 1 was up-regulated at the S1 stage by RR treatment (Figure 3).

### 2.4. Validation of Different Gene Expression Using Real-Time Quantitative PCR (qRT-PCR)

**Figure 4:**
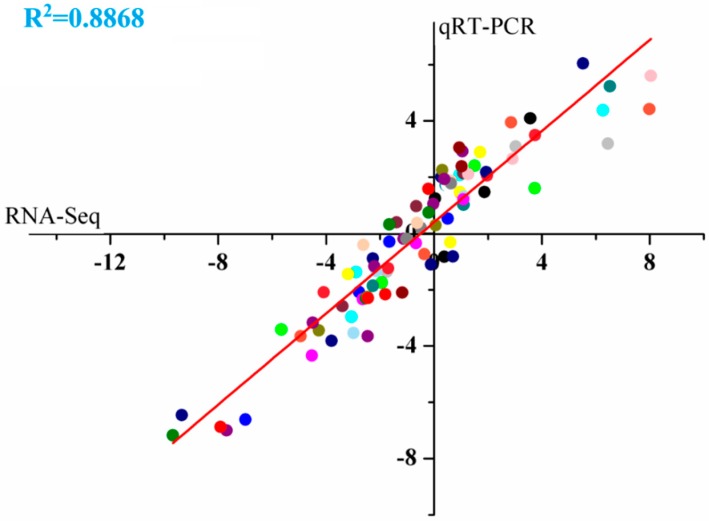
qRT-PCR validation of differential expressed genes between RR treatment and control during the development and ripening of grape berries. Correlation of fold changes obtained by RNA-Seq (x axis) and that obtained by qRT-PCR (y axis).

To confirm the accuracy and reproducibility of the RNA-Seq results, ten differentially expressed genes were selected randomly in the phytohormone biosynthesis and signal transduction pathways for the qRT-PCR analysis (Figure 4), involved in the up-regulated, down-regulated and unaffected genes during the berry development. Linear regression analysis showed an overall correlation coefficient of 0.8868, indicating a good correlation of genes expression results assessed by qRT-PCR and obtained by RNA-Seq, suggesting the reliability of the RNA-Seq data.

## 3. Discussion

Phytohormones play critical roles as regulators of plant growth and development, fruit ripening and physiological responses to stresses. Some phytohormones act as promoters and others as repressors [30]. This is reflected by the contributions of phytohormone biosynthesis and signal transduction pathways, as well as by the diversity of interactions among phytohormones and the level of gene expression to regulate growth responses [2]. Our data indicated that the phytohormone levels changed in responses to RR treatment during grape berry development and ripening, and several transcripts in the phytohormone biosynthesis and signal transduction pathways were involved in the responses.

In the grape berry, it is generally accepted that auxin levels were high after anthesis and then decline to low levels in the ripe berry, but another study showed auxin concentrations relatively constant throughout berry development [31]. In our results, auxin concentrations showed no dramatic changes during the growth processes, and were reduced by RR treatment, consistent with the earlier reports that auxin levels negatively affect stress responses [32]. The growth pattern of grape fruit occurs in two successive sigmoid growth curves separated by a lag phase [33]. Cytokinins are involved in berry set, cell division and ripening inhibition. They also negatively regulate salt and drought stress signaling [34]. RR treatment reduced the cytokinins at two rapid growth phases and accelerated the arrival of concentration peaks. Gibberellins are regulators of a vast number of processes, and are rigidly regulated and moderated by developmental and environmental cues [35]. RR treatment increased or decreased the gibberellins levels at the different stages. The changes of endogenous abscisic acid during grape ripening has been widely reported, the content of which is determined by the dynamic balance between biosynthesis and catabolism [36]. Abscisic acid levels in grape berries decrease after anthesis, but then increase around veraison [37], and was up-regulated in responses to a range of environmental cues, such as water deficit [38,39], temperature [40,41], and salt [42]. We noticed RR treatment induced the transcription factor ABA responsive element binding factor *VIT_18s0001g10450* and increased the abscisic acid concentrations and the 9-*cis*-epoxycarotenoid dioxygenases *VIT_10s0003g03750* and *VIT_19s0093g00550* transcript abundance. Ethylene had been generally considered as a phytohormone that promoted the development and ripening of grape fruits. During grape ripening, ethylene levels slightly increased at the around veraison and the typical respiration peak does not occur [43]. We observed RR treatment increased transcript abundance of genes encoding ACC oxidase and SAM synthetase in ethylene biosynthesis pathway, decreased transcript abundance of genes encoding ethylene receptor, which is a negative regulator of ethylene signaling. Our results suggested that RR increase the ethylene levels during the grape growth, which is consistent with the previous report that ethylene biosynthesis was increased under environmental stresses, such as chilling, flooding, wounding and pathogen attack, and thereby the expression of ethylene-controlled genes were increased. The transcripts in the biosynthesis and signaling pathways were just significantly changed by RR treatment at the earlier stages, indicating that the berry fruit is more sensitive to ethylene in the early developmental stages [44]. In a previous study, the transcript abundance of BR6OX, encoding a negative-feedback enzyme in the brassinosteroid biosynthesis pathway was down-regulated under water deficit [45]. In our results, RR treatment decreased the transcript abundance of brassinosteroid-6-oxidase 2 and this was correlated with an increase in the brassinosteroid contents. In grape berries, jasmonic acid contents increased continuously until 9 weeks post-flowering and then sharply declined to a low level, consistent with the changes of its biosynthesis-related genes [46]. From our results, it can be inferred that RR treatment can increase the jasmonic acid contents during grape berries development and ripening. Jasmonic acid is well known to be increased under wounding, the infection of pathogens, as well as water deficit. This phytohormone was suggested to be involved in the positive response of berry to stresses [47]. Salicylic acid is a phenolic compound produced by plants. Water deficit increased salicylic acid content in pea-sized berries but decreased salicylic acid at the onset of veraison. Previously, salicylic acid application to grape berries at the veraison stage has been shown to induce the expression of genes involved in phenolic biosynthesis [48]. We observed that RR treatment had the same variation tendency about salicylic acid as water deficit in the grape berries during development and ripening. 

Ripening is a complex and highly coordinated developmental process whereby green fruit are converted into a highly palatable, nutritionally rich, and colored fruit [32]. According to the previous studies, abscisic acid, brassinosteroids, and ethylene promote ripening through complex interactions, as well as the role of auxin, cytokinin, and gibberellin as inhibitors of ripening [1,33]. Our results showed RR treatment plays a positive impact on berry ripening through increasing abscisic acid, brassinosteroids, ethylene, jasmonic acid and salicylic acid levels, as well as decreasing auxin, cytokinin, and gibberellin contents.

## 4. Materials and Methods

### 4.1. Materials Collected

This study was carried out during the fruiting season of 2013–2014 in the greenhouse of the orchard of Jinhua Academy of Agricultural Sciences (Zhejiang, China) using three years old plants of table grape “Summer Black” (*V. vinifera* × *V. labrusca*). The plants of RR treatment were planted at 40 cm depth in 100 cm wide ridges isolated with plastic film from outside ground. The plants of the control group were planted in the raise bed (40 cm deep) with the same soil in open ground. The plants in both groups were subjected to the same water and fertilizer managements. Fruit of five different developmental stages were collected in the same day: S1, fruitlet, 15 days after full bloom (DAFB); S2, immature green, 28 DAFB; S3, before veraison, 42 DAFB; S4, veraison, 53 DAFB; S5, fully ripe, 74 DAFB. For each treatment, 10 grape clusters with no evidence of disease or stress symptoms were randomly picked from different plants at each sampling time. All samples were transported to the laboratory immediately. Berries of uniform maturity and without mechanical damage were selected, cut into small pieces, frozen in liquid nitrogen, and then stored at −80 °C for future use. Three biological replicates were performed for each sample.

Different physical properties, including chromatic aberration, total soluble solids (TSSs), total phenolics, total anthocyanins and total procyanidins, were measured throughout the development in “Summer Black” berries with both control and RR treatments. The fruits in RR treatments showed significantly higher TSS level, as well as higher total anthocyanins and total procyanidins during the whole sampling period compared to the control, which had been reported in our previous study [9].

### 4.2. Auxin, Cytokinin, Gibberellin and Brassinosteroid Quantification

For the quantification of auxin, cytokinin, gibberellin and brassinosteroid, 1 g of fresh berries was ground in a mortar and homogenized in extraction solution (9 ml of PBS, pH 7.2–7.4) for enzyme-linked immunosorbent assay (ELISA) [49]. The ELISA procedures were conducted according to the instructions provided by the manufacturer (Enzyme-linked biological technology Co., LTD., Shanghai, China) on the Thermo Labsystems AC8 and Labsystems Multiskan MS 352 (Thermo Labsystems Co., Vantaa, Finland).

### 4.3. Abscisic Acid and Salicylic Acid Quantification

Abscisic acid and salicylic acid were analyzed by UPLC-HRMS. Lyophilized berry powder was extracted with 70% aqueous ethanol (containing 1% formic acid). Chromatographic separation was performed in the UPLC system (Waters Corp., Milford, MA, USA) by an Agilent Eclipse Plus C18 column (4.6 mm × 100 mm, 1.8 μm) with mobile phase A (1% formic acid-water) and mobile phase B (1% formic acid-50% aqueous acetonitrile), with an injection volume of 5 μL and flow rate of 0.75 μL/min. The gradient program was as follows: 0–4 min, 96–92% A; 4–18 min, 92–84% A; 18–22 min, 84–83% A; 22–26 min, 83–79% A; 26–28 min, 79–75% A; 28–34 min, 75–50% A; 34–40 min, 50–5% A; 40–45 min, 5–4% A. UV detector was set at 200–600 nm. Mass spectrometry analysis was carried out using a quadrupole-time-of-flight (AB SCIEX, Triple TOF 5600^plus^ System, Framingham, MA, USA) equipped with electrospray ionization (ESI) source. The optimal MS conditions were: scan range m/z 100–2000; negative ion mode; source voltage was −4500 V and the source temperature 550 °C. The pressure of gas 1 (air) and gas 2 (air) were set to 50 psi, the pressure of curtain gas (N_2_) was set to 30 psi; maximum allowed error was set to ±5 ppm; declustering potential (DP) 100 V, collision energy (CE) 10 V; collision energy of 40 V with a collision energy spread of ±20 V. The exact mass calibration was performed automatically before each analysis employing the automated calibration delivery system. PeakView 1.2.0.3 (AB SCIEX, Framingham, MA, USA) was used for raw data analysis.

### 4.4. RNA Extraction and RNA-Seq

Total RNA was extracted from frozen whole grape berry powder according to our previously published method [50]. After removal of contaminating genomic DNA with a TURBO DNA-free kit (Ambion, Life Technologies, Thermo Fisher Scientific Foster City, CA, USA), the total RNA was quantified using Nanophotometer Pearl (Implen, Inc., WestlakeVillage, CA, USA) before RNA-Seq and real-time PCR analysis. RNA-Seq was carried out with three biological replications. The cDNA libraries were constructed using the TruSeqTM RNA Sample Preparation Kit (Illumina, Inc. San Diego, CA, USA) according to the manufacturer’s instructions. The raw reads were obtained by the Shanghai Majorbio Bio-pharm Biotechnology Co. (Shanghai, China) using Illumina HiSeq™ 2000 with 2 × 100 bp paired-end sequencing. Raw reads were initially processed to get clean reads by removing the adapter and low-quality sequences using the software SeqPrep (https://github.com/jstjohn/SeqPrep). The clean reads were aligned to the reference *V. vinifera* genome (http://www.genoscope.cns.fr/externe/Download/Projets/Projet_ML/data/) [8] using TopHat 2.0.13 software (http://tophat.cbcb.umd.edu/) [51] and the quality was assessed by saturation analysis, duplicate reads analysis and gene coverage analysis by using the RSeQC-2.3.2 program (http://code.google.com/p/rseqc/) [52]. Gene expression values were calculated by the read/fragments per kilobase of exon per million fragments mapped reads (RPKM/FPKM) using Cuffdiff program (http://cufflinks.cbcb.umd.edu/). Differential expression was analyzed according to the count values of each transcript in the two libraries using edgeR software [53]. Genes with a false discovery rate (FDR) < 0.05, and estimated absolute log_2_ fold change (FC) > 1 were used as the thresholds for judging significant difference in transcript expression [54]. KEGG (Kyoto Encyclopedia of Genes and Genomes, http://www.genome.jp/kegg/) pathway analysis was performed using the KEGG function of the blast2go webtool. KEGG pathway analysis of the differentially expressed genes was performed using KOBAS (http://kobas.cbi.pku.edu.cn/home.do) [55].

All the raw sequence data has been deposited at NCBI Sequence Read Archive (SRA) with the accession codes: SRX2234711/ SRR4408346, SRX2234711/ SRR4408347, SRX2234711/ SRR4408413, SRX2234711/ SRR4408414.

### 4.5. Validation of Transcriptome Analysis Using qRT-PCR

For qRT-PCR analyses, gene-specific oligonucleotide primers were designed (Table 2) and the gene specificity of each pair of primers was checked by melting curves and product re-sequencing twice. The glyceraldehyde-3-phosphate dehydrogenase (*GAPDH*) gene was employed as the internal control for calculating relative expression of the mRNA [56]. The sequences of GAPDH primers are described in Table 1. qRT-PCR was performed by FastStart Universal SYBR Green (Roche Diagnostics, Shanghai, China), initiated by 10 min at 95 °C and followed by 40 cycles of 95 °C for 30 s, 60 °C for 30 s, and 72 °C for 10 min, and completed with a melting curve analysis program. The PCR mixture (10 μL total volume) comprised 5 μL of Roche FastStart Universal SYBR Green Master (ROX), 0.75 μL of each primer (10 μmol/L), 0.5 μL of diluted cDNA and 3 μL PCR-grade ddH_2_O. No-template controls and melting curve analysis were included for each gene during each run.

### 4.6. Statistical Analysis

The results are the mean ± standard error (SE) of at least three independent replicates and were analyzed using data processing system SPSS16.0 statistical software package (IBM, Armonk, NY, USA). The statistical significance of differences was determined with *t*-test. Figures were drawn by Origin 8.0 (Microcal Software Inc., Northampton, MA, USA).

## 5. Conclusions

This is the first report on the effects of root restriction on phytohormone changes in grape berry that take place during berry development and ripening. Our transcriptomic analysis demonstrated that 57 of transcripts involved in phytohormone biosynthesis and signaling pathways expressed in berries displayed at least two-fold or greater change in transcript abundance response to RR treatment over the course of five stages of grape berry development and ripening. The content of abscisic acid, brassinosteroids, ethylene, jasmonic acid and salicylic acid levels were increased, and auxin, cytokinin, and gibberellin contents were decreased by RR treatment. Our data suggested that RR accelerated the ripening processes in grape berries. We also noticed a few transcripts in the biosynthesis and signal transduction pathways were not well correlated with the changes in phytohormones, which might be due to complex interactions between the phytohormones.

## Figures and Tables

**Table 1:** Differentially expressed transcripts related to the phytohormone biosynthesis and signaling pathways under RR treatments during grape development (FDR < 0.05 and Log_2_|FC| > 1). FDR, false discovery rate; FC, fold change.

Gene ID	Log2FC					Functional Annotation
	S1RR/S1Control	S2RR/S2Control	S3RR/S3Control	S4RR/S4Control	S5RR/S5Control	
*VIT_04s0044g01740*	−1.02					hydroxymethylglutaryl-CoA reductase
*VIT_06s0004g01510*				1.74		lipoxygenase
*VIT_09s0002g01080*	1.80	−2.50				lipoxygenase
*VIT_02s0087g00910*				−1.88		9-*cis*-epoxycarotenoid dioxygenase
*VIT_10s0003g03750*				1.82		9-*cis*-epoxycarotenoid dioxygenase
*VIT_19s0093g00550*	3.72					9-*cis*-epoxycarotenoid dioxygenase
*VIT_14s0083g01110*				−2.15		brassinosteroid-6-oxidase 2
*VIT_19s0140g00120*	1.15					gibberellin 2-oxidase
*VIT_02s0025g00240*	3.84	2.44				beta-carotene 3-hydroxylase
*VIT_16s0050g01090*	1.59					beta-carotene 3-hydroxylase
*VIT_03s0088g01150*	−1.22					squalene monooxygenase
*VIT_11s0016g02380*	3.15		2.55			aminocyclopropanecarboxylate oxidase
*VIT_12s0059g01380*	1.66					aminocyclopropanecarboxylate oxidase
*VIT_15s0048g00370*	−2.00	−1.73				transketolase
*VIT_08s0007g05000*	2.83					*S*-adenosylmethionine synthetase
*VIT_12s0028g00960*				1.99		phytoene synthase
*VIT_00s0391g00070*				1.30		3-deoxy-7-phosphoheptulonate synthase
*VIT_06s0004g02620*		−2.48	3.77	2.90		phenylalanine ammonia-lyase
*VIT_13s0019g04460*	−1.04		3.99	3.02		phenylalanine ammonia-lyase
*VIT_16s0039g01100*		−3.10				phenylalanine ammonia-lyase
*VIT_16s0039g01110*		−2.64				phenylalanine ammonia-lyase
*VIT_16s0039g01120*		−3.50				phenylalanine ammonia-lyase
*VIT_09s0054g01090*	−1.11					cycloartenol synthase
*VIT_01s0026g00940*	1.96					two-component response regulator ARR-A family
*VIT_08s0007g05390*	1.71					two-component response regulator ARR-A family
*VIT_13s0067g03070*	4.18					two-component response regulator ARR-A family
*VIT_18s0001g10450*	1.27					abscisic acid (ABA) responsive element binding factor
*VIT_05s0049g00510*		−3.69	1.84			ethylene-responsive transcription factor 1
*VIT_07s0005g03230*	−1.66	−4.62				ethylene-responsive transcription factor 1
*VIT_07s0005g03260*	−2.23	−3.89				ethylene-responsive transcription factor 1
*VIT_06s0004g05240*		−2.43				ethylene receptor
*VIT_05s0049g00090*		−1.92				ethylene receptor
*VIT_07s0005g00090*	−2.77					auxin-responsive GH3 gene family
*VIT_07s0104g00930*	1.44					gibberellin receptor GID1
*VIT_05s0020g04670*				−1.81		auxin-responsive protein IAA
*VIT_05s0020g04680*				−1.79		auxin-responsive protein IAA
*VIT_05s0049g01970*				−1.61		auxin-responsive protein IAA
*VIT_07s0141g00270*	3.93					auxin-responsive protein IAA
*VIT_09s0002g04080*				−2.65		auxin-responsive protein IAA
*VIT_09s0002g05160*	1.30					auxin-responsive protein IAA
*VIT_11s0016g04490*	2.32			−1.52		auxin-responsive protein IAA
*VIT_14s0030g02310*	−1.11					auxin-responsive protein IAA
*VIT_01s0146g00480*	1.44					jasmonate ZIM domain-containing protein
*VIT_09s0002g00890*	1.21					jasmonate ZIM domain-containing protein
*VIT_11s0016g00710*	3.03					jasmonate ZIM domain-containing protein
*VIT_06s0004g05460*	2.14					protein phosphatase 2C
*VIT_11s0016g03180*	1.30					protein phosphatase 2C
*VIT_13s0019g02200*	1.67					protein phosphatase 2C
*VIT_16s0050g02680*	8.18		2.78	2.56		protein phosphatase 2C
*VIT_03s0088g00710*	4.35			3.60		pathogenesis-related protein 1
*VIT_03s0088g00810*	2.32					pathogenesis-related protein 1
*VIT_02s0012g01270*	−1.26	−2.19				abscisic acid receptor PYR/PYL family
*VIT_02s0154g00010*	3.61	−3.01				SAUR family protein
*VIT_04s0023g00580*	2.69					SAUR family protein
*VIT_15s0048g00530*	1.51					SAUR family protein
*VIT_16s0098g01150*	1.70					SAUR family protein
*VIT_11s0052g01190*	1.89	−4.88	2.07	−3.95		xyloglucosyl transferase TCH4

**Table 2:** Primers for qRT-PCR.

Gene	Forward Primer (5′ to 3′)	Reverse Primer (5′ to 3′)
*GAPDH*	TGGAGCTGAATTTGTTGT	GTGGAGTTCTGGCTTGTA
*VIT_06s0004g01510*	GACTGGCTTGGTAAAACACTCCT	GCACCAATCTCCCCAAAATC
*VIT_06s0004g05460*	TCGCACTACTTCGGAGTTTATGA	ATCGCATTCTTCCAACCTGAC
*VIT_10s0003g03750*	AACCAGATAACTCAGCCACCG	AAGAAGTGATGCCCAGCGAC
*VIT_11s0016g02380*	CCCTGGAGGATAAAGAAACTGC	TTCTCCAAACCAAGATTCTCACA
*VIT_13s0067g00330*	CTCTACACTTTCGGCGGACAC	CACAGCAGCATCACGGAATC
*VIT_14s0083g01110*	AACATCCAGGAGAAAACCAAAGA	CCAGGAAGGTCAATAGGCAGA
*VIT_15s0048g00370*	TTTAACCCCAAGAACCCCTACT	TGGCAATACCCTGACCAAGAG
*VIT_16s0050g02680*	GAAATGCCAATCTGAGACCGA	CTCCTCCCAATCACCGACAC
*VIT_18s0001g10450*	GGTGAGGGCAGGTGTAGTGAG	AAAATAGGTGATTGCGTGGAAA
*VIT_19s0093g00550*	AACGCTGGGCTCGTCTATTT	CTCCACGTCTGGGGATTTGT
